# Interplay of Physical Activity and Vitamin D Receptor Gene Polymorphism on Bone Mineral Density

**DOI:** 10.2188/jea.11.229

**Published:** 2007-11-30

**Authors:** Ikumi Kitagawa, Yasuo Kitagawa, Teruo Nagaya, Shinkan Tokudome

**Affiliations:** 1 Nagoya Seirei Junior Collage.; 2 Nagoya University Graduate School of Bioagricultural Sciences.; 3 Nagoya City University Medical School.

**Keywords:** bone density, calcium intake, gene polymorphism, physical activity, vitamin D receptor gene

## Abstract

The interplay of physical activity and vitamin D receptor (VDR) gene polymorphism in their effects on bone mineral density (BMD) was studied for 120 Japanese girls aged 18-19 years. BMD at distal radius in the group with the VDR genotype at the *Apa*I site of *Aa* was significantly higher than that in the *aa* group, but this association disappeared in a group having the habit of physical activity. The *Aa* genotype gave a higher BMD than the *aa* genotype only in the group without the habit of physical activity. The habit of physical activity was associated with a higher BMD only in the *aa* genotype group. The similar interplay was observed in the VDR genotype at the *Taq*I site. We thus suggest that physical activity and VDR genotypes affect BMD in independent mechanism to give a saturated level of BMD. Higher dietary calcium intake tended to be associated with higher BMD only in the *Aa* genotype, suggesting that the calcium intake and VDR genotype affect BMD in a synergistic mechanism.

Many twin and family studies suggested that genetic factors account for as much as 80% of the variation in bone mineral density (BMD)^1^^-^^3^^)^. Among genetic factors, the vitamin D receptor (VDR) gene has been considered to be important since its polymorphism accounted for up to 75% of the genetic effects^4^^)^. This VDR theory has been under lively discussion^5^^, ^^6^^)^, and it is now under debate whether the VDR gene can regulate the sensitivity of BMD to environmental factors such as physical activity^7^^)^ and calcium intake^8^^, ^^9^^)^. A large cross-sectional study of 470 healthy premenopausal women aged 44-50 suggested that the inter-individual allelic variation in the VDR gene could modulate the influence of physical activity on BMD^10^^)^. Nonetheless, the effect of the VDR genotype on bone density was of limited importance in the study population composed of physically active women aged 43 to 68 years^11^^)^. Loading a progressive exercise three times a week for 18 months increased the BMD of a population aged 35-45, but the effect was not associated with the VDR genotype^12^^)^. Since these studies indicated a complex gene-environmental interaction, the age of the study population and the index of physical activity need to be carefully controlled in further studies.

By studying a population of Japanese girls with a narrow age window of 18-19 years, we showed recently that the polymorphism at *ApaI* site in VDR gene affects BMD in cooperation with an advanced age at menarche^13^^)^. Our questionnaire about their history of physical activity during the age span of 13-18 years suggested that the habit of physical activity affects BMD in a distinct mechanism from that of the VDR genotypes.

## SUBJECTS AND METHODS

One hundred twenty Japanese girls aged 18-19 years participated in the study. BMD was measured at the distal forearm of the non-dominant arm by dual energy X-ray absorptiometry (DXA, DTX200, Osteometer, Denmark). Weight and height were also measured. The characteristics of this study population were summarized elsewhere^13^^)^. Mean age, height; weight and BMI were 18.4±0.5, 157.5±5.1cm, 52.2±6.5kg and 21.0±2.3kg/m^2^ respectively. The *ApaI* and *TaqI* sites in the VDR gene were detected after amplification by the polymerase chain reaction^13^^)^.

A self-administered questionnaire was used to determine the habitual physical activity and dietary calcium intake. As for the former, we asked about the participant’s choice of either sports or cultural activities for the supplemental class during their junior (13-15 years old) and senior (16-18 years old) high school days. Observing the guidelines of the Ministry of Education, Culture, Sports, Science and Technology of Japan, all Japanese junior and senior high schools have such a supplemental class of at least one school hour per week which lets students enjoy either sports or cultural activities. Since we found that one third of our study population chose only cultural activities throughout the six years of their junior and senior high school period, we took advantage of this choice as an index of their habitual physical activity. Through an interview with such girls, we found that they tend to avoid any kind of sports. These girls were grouped into “No” habitual physical activity and the other girls, who had chosen sports at least for one semester, were grouped into “Yes”. The mechanical strains worked on the skeleton during puberty and adolescence is the best index to know the mechanical effect on bone development but our present study revealed that habit of physical activity based on this questionnaire served as a reliable substitutive index of mechanical effect.

As for dietary calcium intake, we scored our participants’ dietary habits by asking about the frequency with which they ate the following seven food groups; milk, milk products, bean and bean products, meat, fish, dried fish with bone, and green vegetables. In the Japanese dietary situation, these food groups account for about 65% of the calcium intake^14^^)^. The frequency of taking one of these food groups was scored as follows: 4 points indicated taking everyday, 3 points meant 3-6 times/week, 2 points meant 1-2 times/week and 1 point meant never taking. The points were summed to calculate the total scores. An individual with a total score of more than 20 was grouped into “High”, and one with a total score below 19 was grouped into “Low”. No participants had the habit of taking calcium supplements or calcium-added foods.

The data were analyzed using the SPSS software package. Analysis of variance was used to examine the mean difference of BMD, the calcium intake and the habitual physical activity between VDR genotypes. The association of the BMD with physical activity and dietary calcium intake in each VDR genotype was examined by ANCOVA.

## RESULTS

In this study, we analyzed the effects of habitual physical activity on BMD by indexing the choice of sports (“Yes”) or cultural activities (“No”) for the supplemental class during the participant’s high school. Since BMD is a body size dependent measure, we first compared the height, the weight and the lean body mass between “Yes” and “No” subgroups to find no significant difference (Table 1). Same was the case for “High” and “Low” subgroups of dietary calcium intake scored through the habit of eating calcium-containing foods (Table 1). These demonstrated that the habitual physical activity and dietary calcium intake on BMD (described below) were not influenced indirectly by difference of body size.

**Table 1.** Mean weight, height and LBM by physical activity and calcium score.

	n	Mean ± SD		
Habitual physical activity		Weight (kg)	Height (cm)	LBM (kg)
yes	79	51.8±5.7	157.6±4.5	38.1±3.0
no	41	52.9±8.0	157.1±6.1	37.9±3.5

Calcium Score				
high	74	52.6±6.7	156.9±5.0	38.0±3.2
low	46	51.6±6.3	158.4±5.2	38.2±3.2

LBM ; Lean body mass

No significant difference of mean weight, height and LBM between yes and no of habitual physical activity, and between high and low of calcium score.

When the VDR genotype was not considered, habitual physical activity showed only a trend of association of higher BMD at distal radius (“Total” in Table 2). When the participants were grouped into the VDR genotypes of *Aa* and *aa*, the effect of their physical activity became statistically significant. As we have reported previously^13^^)^, the BMD at distal radius was associated with the *ApaI* site polymorphism of the VDR gene in the opposite direction to that reported by Morrison *et al.*^4^^)^. The effect of physical activity was significant in the unfavorable VDR genotype of *aa*, but not in the favorable genotype of *Aa* (Table 2). This implies that physical activity affects BMD only in the population with an unfavorable VDR genotype. Another important observation was that the genotype *Aa* gave a higher BMD than *aa* only in the population with no habitual physical activity. These results show that physical activity and VDR genotypes affect the BMD independently but BMD reaches to a saturated level by either factor.

**Table 2.** Mean bone mineral density by vitamin D receptor genotype and habitual physical activity.

	Mean BMD of radius (g/cm^2^)						

	Habitual past physical activity						
		Total	n	Yes	n	No	n
*ApaI*	AA	0.502±0.061	7	0.509±0.066	6	0.513	1
	Aa	0.515±0.054^a^	53	0.515±0.053	33	0.516±0.057^b^	20
	aa	0.492±0.039	60	0.500±0.037^c^	40	0.476±0.040	20

*TpaI*	TT	0.499±0.048	99	0.506±0.047^d^	67	0.486±0.045^e^	32
	Tt	0.518±0.051	20	0.502±0.047	11	0.538±0.050	9
	tt	0.564	1	0.564	1		0

Total		0.503±0.049	120	0.506±0.047	79	0.496±0.052	41

Statistical analysis was performed according to ANCOVA.

^a^ Differs from the aa (p<0.05)

^b^ Differs from the aa of the ‘No’ group (p<0.05)

^c^ Differs from the ‘No’ group of the aa (p<0.05)

^d^ Differs from the ‘No’ group of the TT (p<0.05)

^e^ Differs from the tt of the ‘No’ group ( p<0.05)

Since the haplotype frequency of *t*/*T* was 0.09/0.91 in the Japanese population^13^^)^, the *TaqI* site polymorphism gave uneven sample numbers for the *tt*, *Tt* and *TT* genotypes and was not statistically very strong. Nevertheless, the BMD of the genotype *Tt* was significantly higher than that of *TT* only in the population with no habitual physical activity. Here again, the effect of physical activity was significant only in the unfavorable VDR genotype of *TT*.

The dietary calcium intake scored through the habit of eating calcium-containing foods suggested a cooperative interplay with the VDR genotype(Table 3). High scores for calcium intake indicate a trend towards higher BMD regardless of the genotype, and this trend was clearer in the *Aa* genotype than in the *aa* genotype.

**Table 3.** Mean BMD by VDR genotype and calcium score.

		Mean BMD of radius (g/cm^2^)					
		Calcium score					
			n	High	n	Low	n
*ApaI*	AA	0.502±0.061	7	0.517±0.051	6	0.413	1
	Aa	0.515±0.054	53	0.523±0.054a	36	0.499±0.054	17
	aa	0.492±0.039	60	0.495±0.035	32	0.488±0.044	28
*TpaI*	TT	0.499±0.048	99	0.506±0.047	58	0.489±0.048	41
	Tt	0.518±0.051	20	0.524±0.050	15	0.502±0.055	5
	tt	0.564	1	0.564	1		0

Total				0.510±0.048	74	0.491±0.048	46

Statistical analysis was performed by ANCOVA.

a ; Differs from aa of High group (p<0.05)

## DISCUSSION

In a survey of 470 healthy premenopausal women aged 44-50, Salamone *et al.* found that the VDR genotype modifies the association between physical activity and BMD at least at the femoral neck^10^^)^. Within the population having the *bb* genotype, which resulted in lower BMD, higher physical activity was associated with a higher femoral neck BMD. The effect of physical activity was less pronounced for the *BB* and *Bb* genotypes. Their pattern was consistent with our results for *ApaI* and *TaqI* sites, suggesting together that the VDR genotype affected the BMD (at least at the femoral neck and distal radius) in an independent mechanism from that of physical activity. A clearer demonstration of the differential effects of VDR genotype and physical activity in our study may be due to the *ApaI* and *TaqI* sites, but it is more likely due to a different way of evaluating the physical activity. As the index of physical activity, Salamone *et al.* estimated the total kilo-calorie expenditure per week as measured by the questionnaire. Since our questionnaire reflected the habit of participants over the period of 13-18 years old, it might be a better index of physical activity during the period of bone development. In a similar study on the interplay of VDR genotype and physical activity, Jarvinen *et al.* loaded the participants aged 35-45 years a progressive exercise three times a week for a period of 18 months, but they did not observe any modification of the association between VDR genotype and BMD through physical activity^12^^)^. Apparently different results suggest that “habit” (over a long period) and “loading” (within a limited period) of physical activity need to be distinguished to evaluate its effect on BMD.

If we can assume that the effect of VDR genotype on BMD is due to the role of VDR in calcium metabolism, the enhanced osteocyte proliferation demonstrated by Chambers *et al.* by mechanical stimulation in rat tail vertebrae may provide an explanation for the differential effects of VDR genotype and physical activity^15^^)^. They showed that mechanical loading induces bone formation through the expression of immediate early genes such as *c-fos* and insulin-like growth factor I in otseocytes^16^^)^. This animal model system suggests that physical activity enhances “osteogenesis” but not “calcification” in the subsequent step of bone formation. This may explain why the effect of physical activity on BMD was clear in the population with unfavorable VDR genotypes of *aa*, *TT* and *bb*. In the favorable VDR genotypes, reduced osteogenesis due to low physical activity may be overcome by the enhancement of calcium metabolism. In the study by Rauch *et al.*^11^^)^, the impact of the VDR genotype was not clear probably because all of their study population consisted of physically active women.
